# Combined effect of triglyceride glucose-body mass index and hypertension on new-onset stroke: evidence from the China health and retirement longitudinal study

**DOI:** 10.3389/fpubh.2024.1432742

**Published:** 2024-10-25

**Authors:** Fucun Ma, Jiaying Hu, Zheng Gao, Xuekai Liu, Mingjian Bai, Guowei Liang

**Affiliations:** Department of Clinical Laboratory, Aerospace Center Hospital, Beijing, China

**Keywords:** triglyceride glucose-body mass index, hypertension, stroke, China health, combined

## Abstract

**Objective:**

The aim of this study is to investigate the combined impact of the triglyceride glucose-body mass index (TyG-BMI) and hypertension on the risk of stroke among the middle-aged and older adult population in China.

**Methods:**

This study included 6,922 participants aged 45 and above from the China Health and Retirement Longitudinal Study, utilizing a multivariate *Cox* proportional hazards regression model to explore the relationship between TyG-BMI, hypertension, and the incidence of new-onset stroke events, as well as conducting Net Reclassification Improvement (NRI) and Integrated Discrimination Improvement (IDI) analyses to evaluate the predictive utility of TyG-BMI.

**Results:**

During a 7-year follow-up period, a total of 401 stroke events were recorded. Compared to patients with lower TyG-BMI (TyG-BMI < 199.74) levels and non-hypertension, those with elevated TyG-BMI levels and non-hypertension had an adjusted hazard ratio (*HR*) and 95% confidence intervals (95%*CI*) were 1.47 (1.05–2.05). The adjusted *HR* and 95%*CI* for the group with lower TyG-BMI levels and hypertension was 2.99 (2.17–4.12), and for those with elevated TyG-BMI levels and hypertension, the adjusted *HR* and 95%*CI* was 3.49 (2.63–4.62). In a multivariate *Cox* proportional hazards regression model, the combination of elevated TyG-BMI levels and hypertension, treated as routine variables, was still significantly associated with the risk of stroke. NRI and IDI analyses showed significant improvements in risk prediction with the inclusion of TyG-BMI. Furthermore, in all subgroup analyses conducted, individuals with elevated TyG-BMI levels and hypertension nearly exhibited the highest risk for incident stroke.

**Conclusion:**

Our study reveals that the combined effect of TyG-BMI and hypertension may increase the risk of incident stroke in the middle-aged and older adult Chinese population. TyG-BMI correlates with comorbid conditions and enhances traditional risk assessment. Future research will require validation through larger sample sizes or diverse populations to further confirm this finding.

## Introduction

Stroke is the second leading cause of death worldwide after heart disease and poses a significant burden on both healthcare and the economy, making it a prominent global health issue (1, 2). Based on figures from the World Health Organization (WHO) and the 2020 Heart Disease and Stroke Statistics report, approximately 7 million individuals globally lost their lives to stroke in 2012, which constituted 11.1% of total deaths (3). Although the age-standardized death rate for stroke has decreased over the past few decades (2), the age-standardized incidence of stroke has increased in China. In China (4), which sees over 2 million new cases annually, stroke tops the list of diseases in terms of the highest loss of disability-adjusted life years (DALYs). The burden is expected to increase further due to an aging population, continued high prevalence of risk factors such as obesity, hypertension, hyperlipidemia, hyperglycemia, and inadequate management. Thus, based on traditional risk factors, we focus on modifiable novel risk factors that are more effective for prevention and potentially widely applicable for the prevention and treatment of stroke.

Insulin resistance (IR) is recognized as an early manifestation of type 2 diabetes and a major pathophysiological pathway in the development of type 2 diabetes (5, 6). It is not only present in type 2 diabetes but also in conditions such as atherosclerosis, hypertension, atrial fibrillation, and coronary heart disease (7–10). Previous studies have indicated that IR is a novel risk factor for stroke and is a key factor in the pathogenesis of a variety of cardiovascular diseases (11, 12). Now, there are many methods to evaluate IR, including Homeostasis Model Assessment of IR (HOMA-IR) has been widely applied and proven to be valid in predicting the occurrence of heart disease and vascular issues (13–16). However, its disadvantage is that patients need to measure fasting insulin levels, making it time-consuming and expensive in daily practice and large epidemiological studies, limiting its clinical application. Recent studies have shown that the Triglyceride Glucose (TyG) index, which is derived from triglyceride (TG) and fasting blood glucose (FBG) levels, can serve as a simple substitute indicator for IR due to its strong correlation with HOMA-IR and better performance in detecting insulin sensitivity (17). This indicator can be obtained from routine clinical laboratory tests and is associated with the occurrence and recurrence of stroke. Experiments have demonstrated that the TyG index is superior to HOMA-IR in predicting stroke (18, 19). A recent study has shown that TyG-BMI can be used as a potential and direct marker of IR. It is calculated as ln [TG (mg/dl) × FBG (mg/dl)/2] × BMI (kg/m^2^) (20). By incorporating BMI into the calculation of the TyG-BMI index, this newly established index may enhance the ability of TyG-BMI to elucidate the impact of obesity on IR (20). The combination of obesity and TyG may prove superior to other alternative markers for the early detection of IR, as obesity is a well-known risk factor for IR (21). Some studies have also indicated that TyG-BMI exhibits better predictive accuracy than TyG in metabolic or cardiovascular diseases (22–24).

Hypertension is the most significant risk factor for cardiovascular disease and also contributes to the risk of stroke (25, 26). It worsens the advancement of atherosclerosis, the primary cause of stroke (27). The China Patient-Centered Cardiac Event Assessment (PEACE) report indicates that around half of the Chinese population aged 35–75 is affected, with 84.2% of stroke survivors having hypertension (28, 29). In recent years, studies have shown a significant correlation between TyG and hypertension. The TyG index is an independent risk factor for new-onset hypertension (30). However, there is a scarcity of literature exploring the relationship between changes in TyG-BMI and hypertension with the incidence of stroke. Few studies have specifically evaluated the cumulative effect of TyG-BMI and hypertension on the risk of new stroke in the general population.

By innovatively assessing the joint influence of the TyG-BMI index and hypertension on stroke risk in the middle-aged and older adult Chinese population, this study aims to develop a more precise predictive model for stroke risk. The findings are expected to inform the development of targeted prevention strategies and contribute to the global understanding of stroke risk factors by providing insights from a population undergoing rapid epidemiological changes.

## Materials and methods

### Study population

The CHARLS is an ongoing nationwide cohort study that uses a multistage clustering sample method to select participants aged ≥45 years from the Chinese population, evaluating their social, economic, and health status (31). A total of 17,708 participants for CHARLS were obtained from 10,257 households within 150 districts and 28 provinces in the baseline survey (2011–2012, Wave 1) (31). CHARLS respondents were followed up every 2 years. The first visit was conducted in 2011–2012 (Wave 1) with 17,708 patients. Subsequently, three the follow-up visits were conducted among survivors in 2013–2014 (Wave 2), 2015–2016 (Wave 3), and 2017–2018 (Wave 4). Data were collected through face-to-face computer-assisted personal interviews. The ethics application for collecting data on human subjects in CHARLS was approved by the Biomedical Ethics Review Committee of Peking University (IRB00001052-11015), and all CHARLS participants provided written consent and informed consent. The details of the CHARLS data are available on its website. Website: http://charls.pku.edu.cn/en.

**Figure 1:**
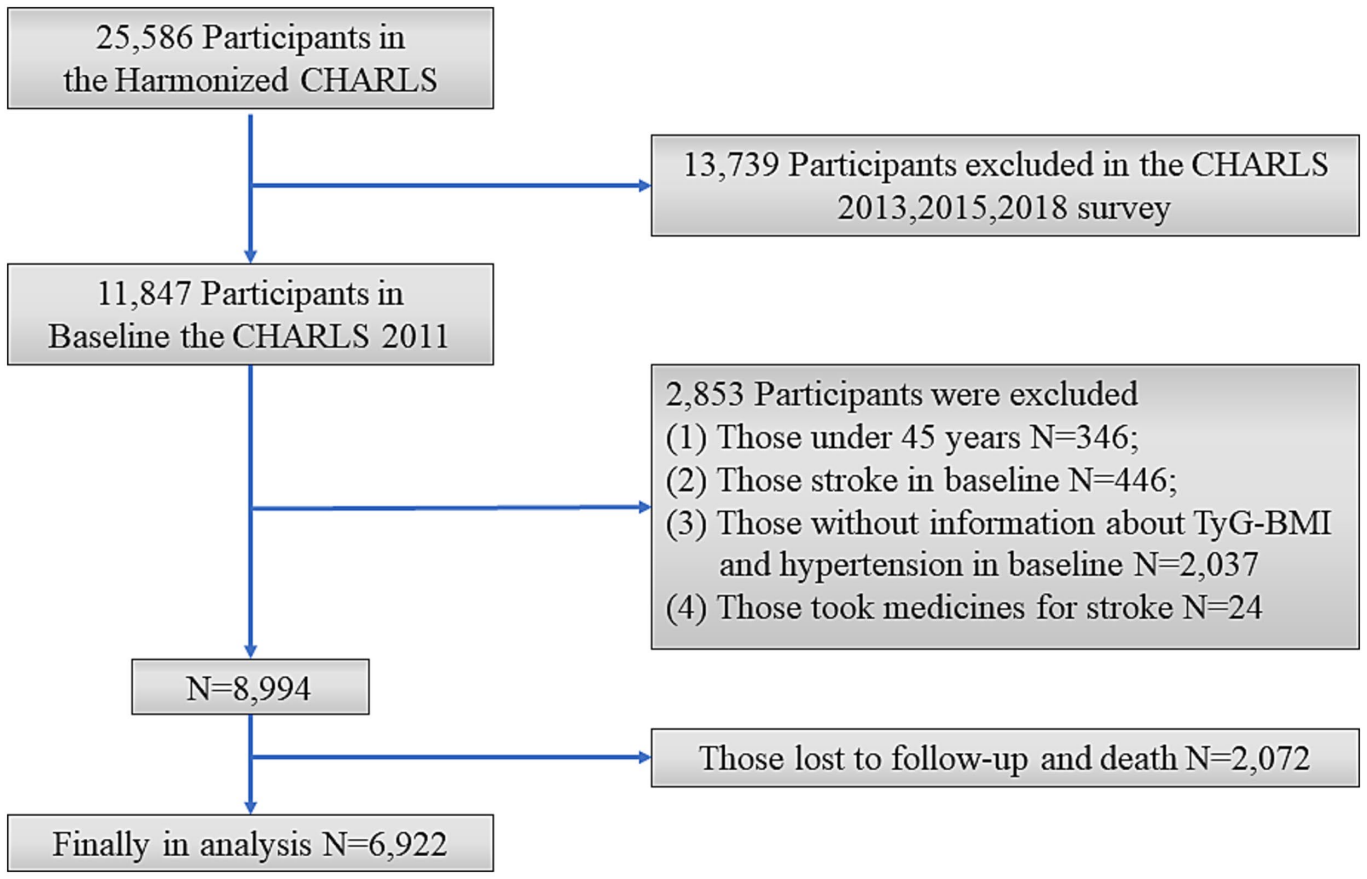
Flow chart of sample selection and the exclusion criteria.

This study excluded individuals who met the following criteria: aged <45 years; had a history of stroke in Wave1; had incomplete information on TyG-BMI and hypertension; took medicines for stroke; with loss to follow-up or death during the follow-up period. The final sample consisted of 6,922 individuals (Figure 1).

### Blood sample collection and measurement

Three tubes of venous blood were collected from each respondent by medically trained staff from the Disease Control and Prevention (CDC), based on a standard protocol. The samples were then promptly transported to the local laboratory and stored at 4°C. The blood samples were centrifuged and stored at-20°C, then transported to the China Center of Disease Control laboratory in Beijing within 2 weeks. Upon arrival, they were stored at-80°C before analysis. FBG and TG were measured by enzymatic colorimetric test.

### The definition of TyG-BMI index and hypertension

The Wave1 National Baseline Blood Data Users’ Guide shows that CHARLS has resoundingly accumulated and analyzed venous blood samples from a large population. The TyG-BMI index was the exposure variable of the present study, computed utilizing FBG, TG and BMI, and the formula is as follows: TyG-BMI index = ln [TG (mg/dl) × FBG (mg/dl)/2] × BMI (kg/m^2^) (20). Height and weight were measured by a trained nurse. BMI was calculated as weight in kilograms divided by height in meters squared. Trained staff used an automated sphygmomanometer (Omron HEM-7200 Monitor) to measure the participants’ blood pressure on the left arm. The bottom edge of the cuff was positioned approximately 0.5 inches above the elbow. Measurements were taken after the participants had been sitting and resting for at least 10 min. Each measurement was separated by 45–60 s, for a total of three measurements. The participants were classified as having hypertension when their systolic blood pressure (SBP) was ≥140 mmHg and/or diastolic blood pressure (DBP) was ≥90 mmHg. Alternatively, participants with a self-reported history of hypertension, as well as those who have used antihypertensive drugs, were also classified as hypertensive (32, 33).

### Ascertainment of new-onset stroke and onset time

The primary outcome of this study was a stroke event. According to previous studies (34, 35), stroke events were determined by self-reports where individuals confirmed having received a diagnosis of stroke from a physician or by answering the following standardized question: “Have you been told by a doctor that you have been diagnosed with a stroke?” Participants who reported a stroke during the follow-up period were classified as having experienced a stroke. The date of stroke diagnosis was recorded as the date between the last interview and the interview where the incident stroke was reported.

### Covariates assessments

In the baseline survey, we collected a range of covariates, including age, sex, self-reported educational level, self-reported marital status, household registration type, self-reported smoking and drinking habits, history of chronic diseases (such as diabetes, cancer, arthritis, dyslipidemia, digestive system diseases, chronic lung diseases, psychological issues, liver disease, kidney disease, memory problems), and physical examination indicators (FBG, waist circumference, TG, C-reactive protein (CRP)), BMI. Educational level was categorized into three tiers: primary school or below, junior high school, and senior high school or above. Household registration was differentiated into agricultural and non-agricultural. Smoking and drinking status were denoted by “yes” or “no.” BMI was calculated by dividing an individual’s weight in kilograms by the square of their height in meters. Self-reported chronic diseases were similarly classified as “yes” or “no.”

### Incremental value of TyG-BMI in cardiovascular risk assessment

To explore whether the TyG-BMI index offers additional discriminatory information beyond traditional risk factors in cardiovascular risk assessment, this study conducted a comprehensive analysis within the CHARLS cohort. The purpose of the analysis was to evaluate if the inclusion of the TyG-BMI index enhances the predictive accuracy of risk among participants stratified by baseline risk levels. Participants were initially stratified by their baseline cardiovascular risk using established criteria, including age, alcohol consumption, waist circumference, CRP, hypertension, cardiovascular disease, and dyslipidemia. The TyG-BMI index was calculated for each participant and incorporated into the risk prediction models for further analysis.

### Statistical analysis

The median TyG-BMI level was 199.74. All participants were divided into four groups based on their hypertension and TyG-BMI levels: TyG-BMI < 199.74 and non-hypertension (Group 1); TyG-BMI ≥ 199.74 and non-hypertension (Group 2); TyG-BMI < 199.74 and hypertension (Group 3); TyG-BMI ≥ 199.74 and hypertension (Group 4).

In the baseline characteristics of the participants, continuous variables that were normally distributed are presented as mean ± standard deviation (*SD*), while those not normally distributed are presented as interquartile range (*IQR*). Categorical variables are expressed as frequencies and percentages. Differences among categorical variables were compared using the Pearson chi-square test, and differences among continuous variables were analyzed using the Kruskal-Wallis test or analysis of variance (ANOVA). *Kaplan–Meier* survival curves and log-rank tests were employed to compare the cumulative risks of stroke across different levels of TyG-BMI and hypertension groups. We utilized a multivariable *Cox* proportional hazards model to estimate the *HR* and their 95% *CI* for the association between elevated TyG-BMI levels, hypertension, and incident stroke.

To evaluate the discriminatory ability of models with and without TyG-BMI, we performed Receiver Operating Characteristic (*ROC*) curve analysis. NRI and IDI (36) were calculated to assess the impact of TyG-BMI on risk reclassification and model discrimination, respectively.

Furthermore, we conducted several sensitivity analyses to validate the robustness of our findings, including different definitions of hypertension, consideration of medication history, and exclusion of individuals receiving baseline hypertension treatment. Subgroup analyses revealed the combined impact of elevated TyG-BMI levels and hypertension on the risk of incident stroke, highlighting particular populations with significant findings. The statistical data analysis software packages *Stata/MP* 18.0 and *R version* 4.4.1 were used for all the data analyses. The statistical significance level was set as 0.05 (two-tailed).

## Results

### Population characteristics

Baseline characteristics of the study population in different groups are presented in Table 1. Out of the 6,922 participants included in the final analysis, the prevalence of new-onset stroke was 5.79% (*N* = 401). Among 6,922 participants, 2,450 (35.39%) individuals had low TyG-BMI level and non-hypertension, 1,727 (24.95%) had elevated TyG-BMI level and non-hypertension, 1,011 (14.61%) had low TyG-BMI level and hypertension, and 1,734 (25.05%) had both elevated TyG-BMI level and hypertension. Statistically significant differences were observed in age, sex, education level, marital status, household registration, drinking and smoking habits, history of diabetes, arthritis, dyslipidemia, digestive system, lung diseases, memory problems, FBG, waist measurement, TG, CRP and BMI baseline indicators among the four subgroups (all *p* < 0.05).

**Table 1:** Characteristics of the study population in four groups in CHARLS.

Variable	Group = 1	Group = 2	Group = 3	Group = 4	*p*-value
No. of subjects	*N* = 2,450	*N* = 1,727	*N* = 1,011	*N* = 1,734	
Age, years	58.15 ± 8.51	55.47 ± 7.63	62.67 ± 9.07	59.13 ± 8.55	<0.001
Sex, *n* (%)					
Male	1,276 (52.1%)	656 (38.0%)	508 (50.2%)	687 (39.6%)	<0.001
Female	1,174 (47.9%)	1,071 (62.0%)	503 (49.8%)	1,047 (60.4%)	
Education level, *n* (%)					
Primary school or below	1,740 (71.0%)	1,098 (63.6%)	813 (80.4%)	1,217 (70.2%)	<0.001
Middle school	472 (19.3%)	415 (24.0%)	148 (14.6%)	342 (19.7%)	
High school or above	238 (9.7%)	214 (12.4%)	50 (4.9%)	175 (10.1%)	
Marital status, *n* (%)					
No	2,435 (99.4%)	1,722 (99.7%)	1,001 (99.0%)	1,729 (99.7%)	0.045
Yes	15 (0.6%)	5 (0.3%)	10 (1.0%)	5 (0.3%)	
Household registration, *n* (%)					
Agricultural	2,189 (89.3%)	1,395 (80.8%)	907 (89.7%)	1,396 (80.6%)	<0.001
Non-agricultural	261 (10.7%)	331 (19.2%)	104 (10.3%)	337 (19.4%)	
Drinking, *n* (%)					
None	1,441 (58.9%)	1,132 (65.6%)	575 (56.9%)	1,098 (63.4%)	<0.001
Yes	1,006 (41.1%)	593 (34.4%)	436 (43.1%)	635 (36.6%)	
Smoking, *n* (%)					
No	1,362 (55.6%)	1,202 (69.6%)	555 (54.9%)	1,171 (67.5%)	<0.001
Yes	1,086 (44.4%)	524 (30.4%)	456 (45.1%)	563 (32.5%)	
Diabetes, *n* (%)	51 (2.1%)	106 (6.2%)	33 (3.3%)	194 (11.3%)	<0.001
Cancer, *n* (%)	20 (0.8%)	13 (0.8%)	5 (0.5%)	16 (0.9%)	0.68
Arthritis, *n* (%)	820 (33.6%)	611 (35.5%)	383 (38.0%)	639 (36.9%)	0.039
Dyslipidemia, *n* (%)	76 (3.2%)	140 (8.3%)	59 (5.9%)	334 (19.7%)	<0.001
Digestive disease, *n* (%)	654 (26.8%)	374 (21.8%)	237 (23.5%)	349 (20.2%)	<0.001
Lung disease, *n* (%)	272 (11.2%)	134 (7.8%)	135 (13.4%)	180 (10.4%)	<0.001
Psych problem, *n* (%)	30 (1.2%)	22 (1.3%)	13 (1.3%)	21 (1.2%)	0.99
Liver disease, *n* (%)	78 (3.2%)	63 (3.7%)	30 (3.0%)	52 (3.0%)	0.69
Kidney disease, *n* (%)	140 (5.7%)	91 (5.3%)	52 (5.2%)	95 (5.5%)	0.91
Memory problem, *n* (%)	19 (0.8%)	5 (0.3%)	15 (1.5%)	23 (1.3%)	<0.001
FBG, mg/dl	101.33 ± 22.28	114.62 ± 39.27	103.48 ± 23.15	120.45 ± 47.46	<0.001
Waist measurement, cm	77.55 ± 9.35	88.49 ± 12.44	79.38 ± 9.50	92.65 ± 11.07	<0.001
TG, mg/dl	80.54 (61.95–107.97)	135.40 (99.12–198.24)	84.07 (64.61–113.28)	144.26 (104.43–209.74)	<0.001
CRP, mg/l	0.75 (0.44–1.53)	1.06 (0.60–2.07)	0.84 (0.47–1.99)	1.38 (0.76–2.70)	<0.001
BMI, kg/m^2^	20.90 (19.45–22.24)	25.39 (24.00–27.18)	21.10 (19.70–22.30)	26.26 (24.50–28.51)	<0.001

Group 1 is TyG-BMI < 199.74 and non-hypertension; group 2 is TyG-BMI ≥ 199.74 and non-hypertension; group 3 is TyG-BMI < 199.74 and hypertension; group 4 is TyG-BMI ≥ 199.74 and hypertension. TyG-BMI, Triglyceride glucose-body mass index; TG, triglycerides; FBG, fasting blood glucose; CRP, C-reactive protein.

### Differences in the risk of new-onset stroke among various groups

**Figure 2:**
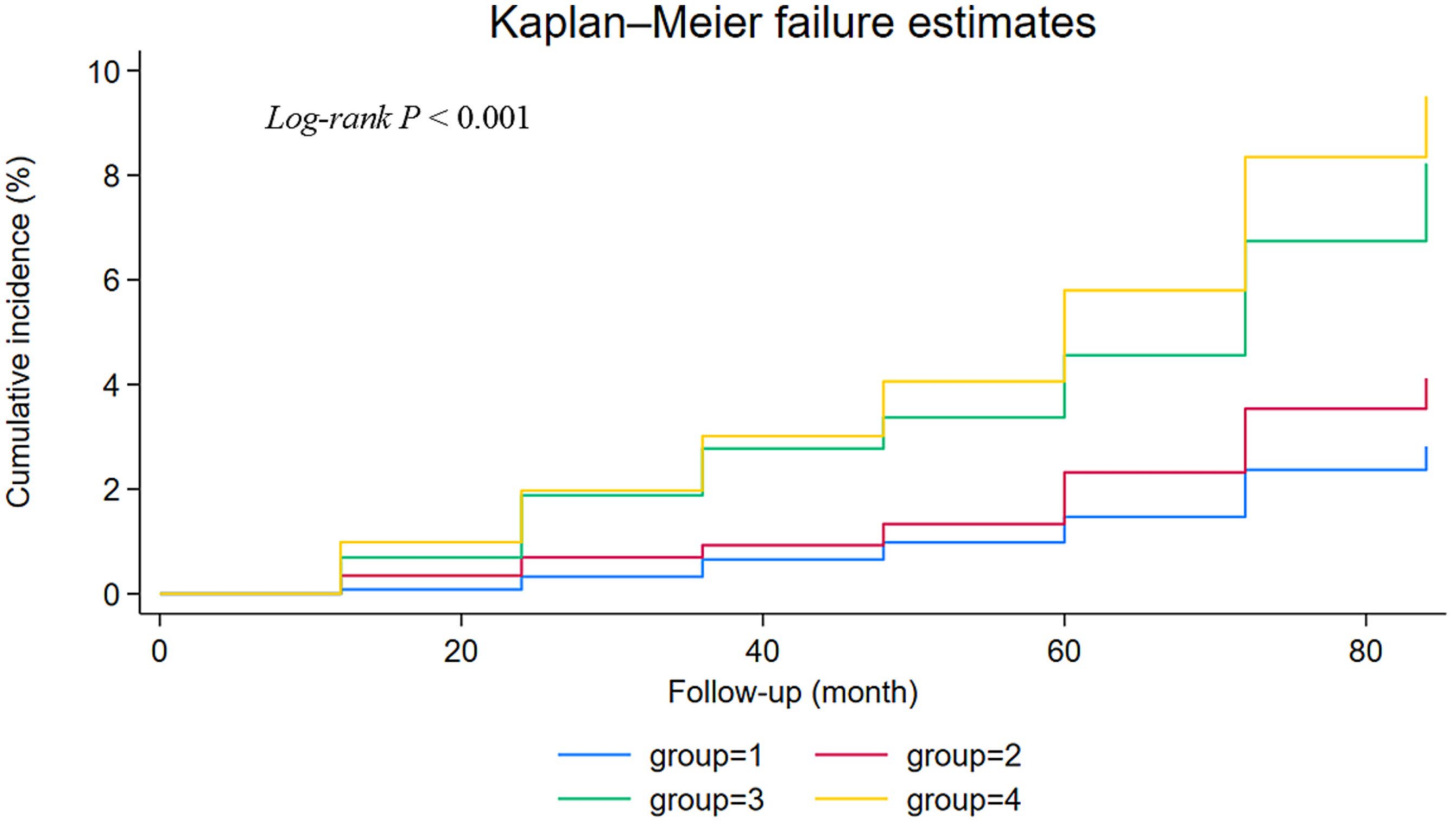
Kaplan–Meier curves for the stroke in four groups in CHARLS.

During the 7-year follow-up, the number of strokes were 70 (2.86%) in group 1, 73 (4.23%) in group 2, 85 (8.41%) in group 3, and 173 (9.98%) in group 4. The *Kaplan–Meier* curves revealed a gradual increase in the cumulative incidence of new-onset stroke from group 1 to group 4. The survival analysis indicated a statistically significant difference among the four groups (*Log-rank* test *p* < 0.001). Individuals in group 4 were found to have a higher risk of new-onset stroke compared to the other three groups (Figure 2). In Model 1, adjusted for age and sex, the risk *HR* (95% *CI*) for stroke in groups 2, 3, and 4 was 1.62 (1.16–2.26), 2.67 (1.93–3.69), and 3.49 (2.63–4.63), respectively, compared to individuals in group 1. After further adjusting for education level, marital status, household status, drinking status, smoking status, FBG, waist measurement, TG, CRP, and BMI in model 2, the *HR* (95% *CI*) for groups 2, 3, and 4 were 1.49 (1.04–2.15), 2.63 (1.90–3.65), and 3.05 (2.17–4.28), respectively. These values still showed a significant association with an increased risk of stroke. Even after adjusting for various chronic disease factors in model 3, the strong association persisted [group 2 (*HR* = 1.49, 95% *CI*: 1.03–2.16), group 3 (*HR* = 2.60, 95% *CI*: 1.87–3.61), group 4 (*HR* = 2.81, 95% *CI*: 1.99–3.96)] (Table 2).

**Table 2:** Association of TyG-BMI and hypertension with new-onset stroke in CHARLS.

New-onset stroke	Group 1	Group 2	Group 3	Group 4	*P* trend
Case, *n* (%)	70 (2.86)	73 (4.23)	85 (8.41)	173 (9.98)	
Model 1^a^	1.00 (ref.)	1.62 (1.16–2.26)	2.67 (1.93–3.69)	3.49 (2.63–4.63)	<0.001
Model 2^b^	1.00 (ref.)	1.49 (1.04–2.15)	2.63 (1.90–3.65)	3.05 (2.17–4.28)	<0.001
Model 3^c^	1.00 (ref.)	1.49 (1.03–2.16)	2.60 (1.87–3.61)	2.81 (1.99–3.96)	<0.001

a Model 1 adjusted for age, sex.

b Model 2 further adjusted for further adjusting for education level, marital status, household status, drinking status, smoking status, FBG, waist measurement, TG, CRP and BMI based on model 1.

c Model 3 further adjusted for diabetes mellitus, cancer, arthritis, dyslipidemia, digestive disease, lung disease, psych problem, liver disease, kidney disease and memory problem based on model 2.

Furthermore, TyG-BMI levels, when considered in isolation, consistently demonstrated a significant correlation with the risk of new-onset stroke across all analytical models (Supplementary Table S1). Similarly, hypertension, when examined alone, was found to be significantly linked to the risk of new-onset stroke in every model analyzed (Supplementary Table S2). However, the risk of new-onset stroke associated with the combined presence of TyG-BMI levels and hypertension was found to be greater than the risk attributable to either TyG-BMI levels or hypertension alone.

### TyG-BMI index association with comorbidities: diabetes and cardiovascular diseases

*Cox* proportional hazards regression analyses were conducted to assess the association between TyG-BMI quartiles and the risk of diabetes and cardiovascular diseases (CVD). Supplementary Tables S3, S4 present the multivariate-adjusted *HR* (95% *CI*) for both outcomes.

### Added value of TyG-BMI to traditional cardiovascular risk assessment

To determine if TyG-BMI provides incremental value over traditional cardiovascular risk factors, we initially compared the discriminatory power of models with and without TyG-BMI using Receiver Operating Characteristic (*ROC*) curve analysis. The area under the *ROC* curve (AUC) was calculated to assess the models’ ability to distinguish between participants who developed stroke and those who did not. Although the addition of TyG-BMI showed a trend toward improved discrimination, the difference was not statistically significant (Supplementary Figure S1), warranting further exploration of its potential value. Subsequently, we conducted NRI and IDI analyses to more thoroughly evaluate the incremental value of TyG-BMI in predicting new strokes. The NRI analysis revealed significant reclassification of individuals into the appropriate risk categories, with an NRI estimate of 0.1589 (95% *CI*: 0.0625 to 0.2611). This indicates a substantial net reclassification of stroke risk upon incorporating TyG-BMI into the risk assessment model. The direction of reclassification was characterized by a NRI+ of 0.0670 (95% *CI*: −0.0112 to 0.1429) and a NRI-of 0.0919 (95% *CI*: 0.0475 to 0.1215), suggesting a shift in risk categories for a portion of the study population. Furthermore, the IDI analysis demonstrated a significant enhancement in the model’s predictive accuracy with the inclusion of TyG-BMI, with an IDI estimate of 0.089 (95% *CI*: 0.000 to 0.167, *p* = 0.040). This signifies that the model’s ability to distinguish between stroke cases and non-cases was notably strengthened by integrating TyG-BMI. In summary, both the NRI and IDI analyses consistently indicate that integrating TyG-BMI into conventional risk assessment models not only refines risk stratification but also significantly enhances the model’s predictive accuracy for stroke. These findings highlight the potential of TyG-BMI as a valuable addition to traditional cardiovascular risk factors, providing a more nuanced approach to identifying individuals at higher risk of stroke.

### Sensitivity and subgroup analysis

Our team carried out an in-depth series of sensitivity analyses, and the results consistently revealed a resilient synergistic effect of TyG-BMI metrics and hypertension on the incidence of new-onset strokes. Conducting sensitivity analyses based on different definitions of hypertension (Supplementary Table S5). Upon further adjustment for medications use for hypertension, diabetes, and dyslipidemia (Supplementary Table S6). Following the exclusion of individuals receiving treatment for hypertension at baseline, a sensitivity analysis was conducted to assess the association between TyG-BMI levels and hypertension with new-onset stroke (Supplementary Table S7). Subgroup analysis indicated that after adjusting for demographic characteristics, lifestyle factors, anthropometric indices, and chronic diseases, individuals with elevated TyG-BMI levels and hypertension were at the greatest risk for incident stroke. The risk of new-onset stroke was particularly high in individuals age ≥ 60 years (*HR* = 2.48, 95% *CI*: 1.59–3.87), female (*HR* = 2.62, 95% *CI*: 1.64–4.21), those with a middle school level (*HR* = 7.89, 95% *CI*: 2.33–26.73), smokers (*HR* = 2.59, 95% *CI*: 1.54–4.37) and alcohol consumers (*HR* = 2.91, 95% *CI*: 1.68–5.02). Notably non-agricultural workers were at a higher risk (*HR* = 4.77, 95% *CI*: 1.65–13.77). Additionally, those with a BMI < 24 kg/m^2^ (*HR* = 2.54, 95% *CI*: 1.56–4.15), FBG ≥ 126.00 mg/dL (*HR* = 4.49, 95% *CI*: 1.42–14.22), waist measurement <87.50 cm (*HR* = 2.44, 95% *CI*: 1.53–3.89), CRP < 3 mg/L (*HR* = 2.84, 95% *CI*: 1.97–4.11), those without dyslipidemia (*HR* = 2.95, 95% *CI*: 2.06–4.22), and non-arthritis (*HR* = 3.87, 95% *CI*: 2.53–5.93) (Supplementary Figure S2). Significant interactions between the subgroup with elevated TyG-BMI levels and hypertension and the aforementioned subgroups were also observed (Table 3).

**Table 3:** Subgroup analysis of *HR* (95% *CI*) of TyG-BMI level and hypertension for Stroke.

Characteristics	Group 1	Group 2	Group 3	Group 4	*p* value	*P*_interaction_
Age, years						
<60	1.00 (ref.)	1.76 (1.01–3.09)	3.85 (2.23–6.65)	2.66 (2.07–6.48)	<0.001	<0.001
≧60	1.00 (ref.)	1.32 (0.77–2.24)	2.10 (1.40–3.15)	2.48 (1.59–3.87)	<0.001	
Sex						
Male	1.00 (ref.)	1.28 (0.73–2.25)	2.75 (1.76–4.29)	2.51 (1.49–4.23)	<0.001	<0.001
Female	1.00 (ref.)	1.44 (0.87–2.36)	2.45 (1.51–3.98)	2.62 (1.64–4.21)	<0.001	
Education level						
Primary school or below	1.00 (ref.)	1.31 (0.86–2.00)	2.20 (1.54–3.15)	2.54 (1.75–3.70)	<0.001	<0.001
Middle school	1.00 (ref.)	4.85 (1.46–16.11)	7.30 (2.27–23.51)	7.89 (2.33–26.73)	0.003	
High school or above	1.00 (ref.)	0.58 (0.15–2.27)	4.27 (1.19–15.36)	1.15 (0.30–4.35)	0.022	
Household registration						
Agricultural	1.00 (ref.)	1.52 (1.03–2.25)	2.39 (1.68–3.39)	2.71 (1.88–3.89)	<0.001	<0.001
Non-agricultural	1.00 (ref.)	1.77 (0.58–5.35)	4.54 (1.67–12.33)	4.77 (1.65–13.77)	0.001	
Drinking						
None	1.00 (ref.)	1.36 (0.84–2.21)	2.77 (1.77–4.36)	2.58 (1.63–4.08)	<0.001	<0.001
Yes	1.00 (ref.)	1.56 (0.86–2.81)	2.52 (1.56–4.07)	2.91 (1.68–5.02)	<0.001	
Smoking						
No	1.00 (ref.)	1.58 (0.99–2.52)	2.54 (1.59–4.05)	2.94 (1.88–4.59)	<0.001	<0.001
Yes	1.00 (ref.)	1.28 (0.71–2.31)	2.66 (1.67–4.23)	2.59 (1.54–4.37)	<0.001	
Diabetes						
No	1.00 (ref.)	1.47 (1.01–2.15)	2.68 (1.92–3.73)	2.63 (1.84–3.74)	<0.001	0.194
Yes	1.00 (ref.)	1.12 (0.19–6.57)	0.83 (0.07–9.41)	4.05 (0.86–19.24)	0.044	
Arthritis						
No	1.00 (ref.)	1.75 (1.10–2.80)	2.63 (1.71–4.06)	3.87 (2.53–5.93)	<0.001	0.027
Yes	1.00 (ref.)	1.03 (0.56–1.89)	2.45 (1.48–4.06)	1.46 (0.80–2.67)	0.002	
Dyslipidemia						
No	1.00 (ref.)	1.63 (1.12–2.39)	2.59 (1.84–3.65)	2.95 (2.06–4.22)	<0.001	<0.001
Yes	1.00 (ref.)	0.53 (0.13–2.12)	2.35 (0.70–7.94)	1.26 (0.39–4.02)	0.097	
Digestive disease						
No	1.00 (ref.)	1.81 (1.18–2.77)	3.15 (2.13–4.65)	3.56 (2.40–5.29)	<0.001	0.554
Yes	1.00 (ref.)	0.83 (0.38–1.79)	1.55 (0.81–2.96)	1.30 (0.61–2.76)	0.310	
Lung disease						
No	1.00 (ref.)	1.48 (1.01–2.17)	2.51 (1.77–3.57)	2.83 (1.98–4.04)	<0.001	0.366
Yes	1.00 (ref.)	1.55 (0.41–5.77)	2.84 (1.10–7.33)	2.17 (0.68–6.93)	0.171	
Liver disease						
No	1.00 (ref.)	1.49 (1.03–2.16)	2.58 (1.85–3.61)	2.84 (2.01–4.00)	<0.001	0.919
Yes	1.00 (ref.)	0.96 (0.10–9.55)	4.72 (0.79–28.24)	1.99 (0.17–23.01)	0.285	
Kidney disease						
No	1.00 (ref.)	1.59 (1.09–2.31)	2.68 (1.91–3.77)	2.89 (2.04–4.10)	<0.001	0.441
Yes	1.00 (ref.)	0.50 (0.08–3.13)	1.73 (0.46–6.46)	1.83 (0.40–8.28)	0.343	
FBG, mg/dl						
<126.00	1.00 (ref.)	1.39 (0.94–2.06)	2.51 (1.78–3.53)	2.46 (1.70–3.56)	<0.001	<0.001
≧126.00	1.00 (ref.)	1.62 (0.48–5.51)	3.42 (1.00–11.65)	4.49 (1.42–14.22)	0.006	
Waist measurement, cm						
<87.50	1.00 (ref.)	1.12 (0.65–1.94)	2.44 (1.70–3.51)	2.44 (1.53–3.89)	<0.001	<0.001
≧87.50	1.00 (ref.)	1.12 (0.57–2.23)	2.14 (0.98–4.68)	2.06 (1.07–3.97)	0.001	
CRP, mg/l						
<3	1.00 (ref.)	1.41 (0.95–2.10)	2.46 (1.71–3.53)	2.84 (1.97–4.11)	<0.001	<0.001
≧3	1.00 (ref.)	1.71 (0.62–4.73)	3.38 (1.49–7.66)	2.33 (0.91–5.97)	0.028	
BMI, kg/m^2^						
<24	1.00 (ref.)	1.41 (0.81–2.45)	2.51 (1.79–3.52)	2.54 (1.56–4.15)	<0.001	0.006
≧24	1.00 (ref.)	2.02 (0.49–8.32)	3.68 (0.61–22.07)	3.85 (0.94–15.70)	<0.001	

In the multivariate models, confounding factors such as age, sex, education level, household registration, drinking, smoking, chronic diseases (diabetes, arthritis, dyslipidemia, digestive disease, lung disease, liver disease, and kidney disease), FBG, waist measurement, CRP, and BMI were included as a subgroup variable.

This study categorized participants into quartiles based on their TyG-BMI index to investigate its association with the risk of stroke. In the non-hypertensive population, we observed a significant increase in the risk of incident stroke with higher TyG-BMI index levels. Specifically, individuals in the fourth quartile (Q4) had the highest risk of stroke compared to those in the first quartile (Q1), with a *HR* of 3.32, a 95% *CI* of 1.30–8.49 (*p* < 0.05). However, in the hypertensive population, the relationship between TyG-BMI index and stroke risk was not significant, with a *HR* of 1.02 for the Q4 group, a 95% *CI* of 0.56–1.87 (*p* < 0.05). Furthermore, we assessed the interactive effect of TyG-BMI index with hypertension status on stroke risk. The analysis revealed a highly significant *p*-value for the interaction term (*P* for interaction <0.001), indicating a marked difference in the impact of the TyG-BMI index on stroke risk between hypertensive and non-hypertensive individuals.

## Discussion

In an extensive, nationwide prospective cohort study encompassing adults over the age of 45, a significant synergistic escalation in stroke risk was observed with concurrent elevated TyG-BMI levels and hypertension. This amplified risk significantly surpassed that attributable to either condition in isolation and remained robust after accounting for a range of other confounding variables, including demographic traits, lifestyle elements, anthropometric indicators, and the presence of chronic diseases. Our findings revealed that individuals with elevated TyG-BMI levels and hypertension exhibited the highest risk of new-onset stroke (*HR* = 3.05, 95% *CI*: 2.17–4.28) compared to those with low TyG-BMI levels and without hypertension. Moreover, both TyG-BMI levels and hypertension were significantly correlated with an increased risk of new-onset stroke when considered individually, but their combined effect was even more pronounced. Further analysis demonstrated that TyG-BMI provides incremental predictive value over traditional cardiovascular risk factors. Incorporating TyG-BMI into risk assessment models significantly enhanced the models’ predictive accuracy for stroke, as evidenced by both NRI and IDI analyses. Sensitivity and subgroup analyses consistently confirmed the synergistic effect of TyG-BMI and hypertension on new-onset stroke, particularly among individuals 60 years of age or older, females, those with lower education levels, smokers, alcohol consumers, individuals with lower BMI, elevated fasting blood glucose, and non-arthritis patients. In summary, our research underscores the importance of considering the combined effects of TyG-BMI and hypertension in stroke prevention and suggests that TyG-BMI could serve as a valuable addition to traditional cardiovascular risk assessment tools.

Building on this practical application, our findings also provide significant insights into the association between TyG-BMI and the presence of comorbidities such as diabetes and cardiovascular diseases. As detailed in Supplementary Tables S3, S4, our *Cox* proportional hazards regression analyses demonstrate a clear trend of increasing risk with higher quartiles of TyG-BMI. This association underscores the importance of considering TyG-BMI alongside traditional risk factors when assessing comorbidity signatures in patient cohorts. Our results are in line with recent studies that have revealed a strong correlation between TyG-BMI and conditions such as prehypertension, non-alcoholic fatty liver disease (NAFLD), and stroke (37–40), further validating the significance of TyG-BMI in predicting adverse health outcomes.

Moreover, our investigation into the incremental value of TyG-BMI over traditional cardiovascular risk factors reveals that while the addition of TyG-BMI to predictive models showed a trend toward better discrimination, this was not statistically significant in *ROC* curve analysis. However, more in-depth analyses using NRI and IDI metrics indicate that TyG-BMI does provide substantial added value in refining risk stratification and enhancing predictive accuracy for stroke. This suggests that integrating TyG-BMI into existing risk assessment models could lead to more nuanced and effective strategies for stroke prevention. These findings are consistent with recent research that has established the TyG-BMI index as a reliable indicator for evaluating insulin resistance and associated metabolic diseases (20), and its potential to serve as an emerging clinical marker for the early diagnosis and differentiation of hypertension.

The TyG-BMI index, first reported by Professor Er in 2016, represents the product of TyG and BMI (20). This combination reflects critical clinical indicators such as blood glucose, blood lipids, and BMI, providing a more comprehensive reflection of IR than the TyG index alone. Recent studies have revealed a strong correlation between TyG-BMI and conditions such as prehypertension, NAFLD, and stroke (38, 41, 42). Hypertension is a significant modifiable risk factor for overall morbidity and mortality worldwide and is associated with an increased risk of cardiovascular diseases (CVD) (43). The relationship between blood pressure and the elevated risk of CVD is graded and continuous, with the risk increasing progressively from levels as low as 115/75 mmHg, which is considered within the normal range (44). This study utilized the baseline data from the 2011 China Health and Retirement Longitudinal Study (CHARLS), encompassing 6,922 participants, of whom 2,745 were hypertensive. This translates to a hypertension prevalence of approximately 39.66% within our studied older adult population, closely aligning with the 43.10% prevalence reported among middle-aged and older adult individuals in the 2015 CHARLS data. The high prevalence rate underscores the widespread nature of hypertension within the Chinese middle-aged and older adult demographic, which may significantly influence our research findings. Prior reports have indicated a significant correlation between the TyG-BMI index and the incidence of hypertension (45, 46). The TyG-BMI index may serve as an emerging clinical indicator for the early diagnosis and differentiation of hypertension. Our study’s findings underscore the potential of the TyG-BMI index to assess stroke risk, particularly in the non-hypertensive population. The observed interaction between TyG-BMI and hypertension status, with a highly significant *P* for interaction <0.001, suggests that the TyG-BMI index’s impact on stroke risk is not uniform across different levels of blood pressure. This interaction highlights the importance of considering an individual’s blood pressure status when evaluating the risk associated with a high TyG-BMI index. Notably, in our study, participants with elevated TyG-BMI levels and hypertension had the highest cumulative incidence of stroke events (Figure 2), indicating that the coexistence of elevated TyG-BMI levels and hypertension poses a greater risk of stroke than either factor alone. Therefore, our research focuses on the combined effect of TyG-BMI and hypertension, which may increase the risk of stroke among middle-aged and older adult populations in China. This combined effect could have significant implications for the development of targeted prevention and treatment strategies, emphasizing the need to address both hypertension and metabolic indicators like TyG-BMI in stroke risk reduction efforts.

The mechanisms underlying the association between elevated TyG-BMI levels and hypertension with the risk of stroke are not fully understood, but several plausible explanations can be posited to account for the potential underlying pathways. The association between TyG-BMI and stroke risk could be linked to the measure of IR (38). IR is characterized as a state where the physiological impact of insulin falls short of the anticipated response within the context of experimental or clinical scenarios. IR plays a role in different tissues depending on their physiological and metabolic functions. Due to the high metabolic demands of IR, it significantly affects skeletal muscle, adipocytes (fat cells), and hepatic tissue, which are also the primary targets for intracellular glucose transport and glucolipid metabolism (47). In skeletal muscle, IR leads to impaired glycogen synthesis. When glycogen synthesis is compromised, the muscle is unable to effectively store glucose, resulting in elevated blood glucose levels. IR also affects the activity of lipoprotein lipase (LPL) in adipocytes. When LPL activity is inhibited, the metabolism of lipids is impacted, which may lead to increased levels of TG in the blood. IR is associated with an increased release of inflammatory cytokines, such as Interleukin-6 (IL-6), Tumor Necrosis Factor *α* (TNF-α), and Leptin, which may be overproduced under conditions of IR. The increase in these inflammatory cytokines can lead to a state of chronic low-grade inflammation, which is associated with the development of various metabolic diseases, including Type 2 diabetes, cardiovascular diseases, and obesity (48–50). Under conditions of IR, the function of endothelial cells may be compromised, leading to a reduction in the production of nitric oxide, and the vascular endothelial cells may release more procoagulant factors, such as tissue factor and von Willebrand factor. Due to the decrease in nitric oxide and the increase in procoagulant factors, platelet aggregation occurs, forming thrombi that can lead to arterial stenosis or occlusion, thereby causing a stroke (48). The primary mechanism linking TyG-BMI to hypertension is likely related to the role of obesity and IR in the development and progression of hypertension (45). Obesity is closely linked to a state of chronic inflammation and oxidative stress in the body. In the state of obesity, certain inflammatory factors and oxidative stress products secreted by adipose tissue can directly or indirectly affect the activity of the Renin-Angiotensin-Aldosterone System (RAAS) and the Sympathetic Nervous System (SNS) (51, 52), leading to an increase in blood pressure. Additionally, fatty acids and inflammatory mediators secreted by adipose tissue can affect the transmission of insulin signals, thereby exacerbating IR and directly or indirectly interfering with the action of insulin. In addition, IR has detrimental effects on various tissue matrices (such as the kidneys), thereby affecting blood pressure regulation (45). Therefore, the potential interaction between TyG-BMI and hypertension may be an important mechanism contributing to the increased risk of stroke.

In this study, we have made a series of significant findings that offer us a fresh perspective for understanding the risks associated with stroke. Firstly, our team innovatively combined TyG-BMI and hypertension as a single composite variable to assess its correlation with stroke risk. The results clearly demonstrated that the combined effect of TyG-BMI and hypertension significantly elevates an individual’s risk of stroke. Secondly, by employing K-M survival curves and *Cox* regression models, we further unveiled the pronounced impact of the combined TyG-BMI and hypertension on stroke risk. Notably, individuals with elevated TyG-BMI levels and hypertension exhibited a significantly increased risk of stroke, an association that remained robust even after adjusting for potential confounding factors. Moreover, through a series of sensitivity analyses, such as refining the definition of hypertension, considering medication history, and excluding individuals receiving baseline hypertension treatment, we further confirmed the universality and stability of this relationship. In subgroup analyses, we specifically highlighted that individual with elevated TyG-BMI levels and hypertension accounted for a substantial proportion of new stroke cases. Particularly, we identified that those aged ≥60, female, non-agricultural, with a middle school education level, who smoke and drink, with a BMI < 24 kg/m^2^, FBG ≥ 126.00 mg/dL, waist measurement <87.50 cm, CRP < 3 mg/L, those without dyslipidemia and non-arthritis patients, are at a particularly heightened risk for stroke. These findings not only substantiate the reliability of our research outcomes but also underscore the synergistic role of TyG-BMI and hypertension as a pivotal risk factor for stroke. This discovery paves the way for novel preventative and therapeutic strategies against stroke, contributing to the enhancement of patient health outcomes and quality of life.

The latest edition of the “2022 AHA Heart Disease and Stroke Statistics Report” presents a forecast indicating that by 2030, approximately 3.4 million US adults (aged 18 and over, representing 3.9% of the total adult population) will be affected by stroke, marking a 20.5% increase in prevalence compared to 2012 (53). Stroke has emerged as one of the leading causes of death and disability worldwide, with the associated costs of post-stroke care being exceedingly high. In light of this, the prevention and management of high-risk groups for stroke become exceedingly critical. Building on previous research (38), this study further explores the combined impact of TyG-BMI and hypertension on the risk of stroke, aiming to deepen our understanding of stroke prevention. Our research confirms that obtaining individual TyG-BMI and hypertension data through straightforward testing methods can effectively prevent the occurrence of stroke. The findings of this study have a significant direct impact on clinical practice, particularly in the early identification and management of stroke risk. Our results emphasize the importance of clinicians in large comprehensive hospitals and community health care facilities being able to easily access key indicators of patients, such as blood lipids, blood sugar, BMI, and blood pressure. These data are crucial for identifying individuals at high risk of stroke and form the basis for timely preventive and control measures, thereby helping to reduce the incidence of stroke. By incorporating TyG-BMI assessment and blood pressure monitoring into routine health evaluations, healthcare providers can more accurately stratify individuals based on their risk profiles. This stratification method not only improves the accuracy of identifying high-risk populations but also provides a basis for implementing personalized prevention strategies, including lifestyle adjustments and pharmacological treatments. These strategies are vital for reducing the risk of stroke, especially in middle-aged and older adult populations where regular health screenings and ongoing monitoring of metabolic health indicators are particularly important.

This study draws conclusions based on the analysis of data from the CHARLS database, which is highly regarded for its representativeness, rigorous design, comprehensive content, and broad application scope. However, there are certain limitations to our research as well. Firstly, establishing the critical value for the TyG-BMI index is of paramount importance. Given that there is currently no standardized threshold to define elevated TyG-BMI levels, the critical value utilized in this study references the median value employed in other research (38) as a benchmark. Secondly, it is important to note that our analysis did not differentiate between ischemic and hemorrhagic strokes, a limitation inherent to the CHARLS database used for this study. While this approach allowed us to assess the collective impact of elevated TyG-BMI levels and hypertension on stroke risk, we acknowledge the value of distinguishing between stroke subtypes for a more nuanced understanding. Future research endeavors will prioritize the inclusion of stroke type classification to enhance the specificity and applicability of our findings. Furthermore, the determination of stroke in this study was based on the self-report of participants or information provided by their physicians, which to some extent introduces the risk of information bias. However, the study also found a high degree of concordance between participant self-reports and physician diagnoses (54, 55), indicating good reliability and validity. Ultimately, it is important to recognize that the CHARLS database focuses on individuals in China who are 45 years of age or older, which means that our research findings may not be generalizable to other age groups or populations with different backgrounds. In the future, we recommend corroborating these results by conducting joint validations with databases from other demographic groups to ascertain the generalizability of our findings. Furthermore, given that our study employs an observational analytical approach, it may be subject to potential biases and confounding factors that were not accounted for.

## Conclusion

In summary, our research findings reveal that in the Chinese middle-aged and older adult population, the coexistence of elevated TyG-BMI levels and hypertension significantly increases the risk of stroke. This discovery underscores the importance of screening for elevated TyG-BMI levels and hypertension among individuals over the age of 45 and suggests that implementing appropriate intervention measures could be significantly beneficial for stroke prevention. In future studies, we aim to further elucidate the biological mechanisms underlying these associations and enhance the predictive power of TyG-BMI and hypertension for stroke risk by extending the follow-up period.

## Data Availability

The raw data supporting the conclusions of this article will be made available by the authors, without undue reservation.

## References

[1] Mozaffarian D Benjamin EJ Go AS Arnett DK Blaha MJ Cushman M. Heart disease and stroke statistics—2015 update. Circulation. (2015) 131:e29–e322. doi: 10.1161/cir.0000000000000152, PMID: 25520374

[2] Feigin VL Nichols E Alam T Bannick MS Beghi E Blake N. Global, regional, and national burden of neurological disorders, 1990–2016: a systematic analysis for the global burden of disease study 2016. Lancet Neurol. (2019) 18:459–80. doi: 10.1016/s1474-4422(18)30499-x, PMID: 30879893 PMC6459001

[3] WHO. WHO methods and data sources for country-level causes of death 2000–2012. Geneva: WHO (2014).

[4] Wu S Wu B Liu M Chen Z Wang W Anderson CS. Stroke in China: advances and challenges in epidemiology, prevention, and management. Lancet Neurol. (2019) 18:394–405. doi: 10.1016/s1474-4422(18)30500-3, PMID: 30878104

[5] Kahn SE. The relative contributions of insulin resistance and beta-cell dysfunction to the pathophysiology of type 2 diabetes. Diabetologia. (2003) 46:3–19. doi: 10.1007/s00125-002-1009-0, PMID: 12637977

[6] Warram JH Martin BC Krolewski AS Soeldner JS Kahn CR. Slow glucose removal rate and hyperinsulinemia precede the development of type II diabetes in the offspring of diabetic parents. Ann Intern Med. (1990) 113:909–15. doi: 10.7326/0003-4819-113-12-909, PMID: 2240915

[7] Yang J Tang Y-D Zheng Y Li C Zhou Q Gao J. The impact of the triglyceride-glucose index on poor prognosis in NonDiabetic patients undergoing percutaneous coronary intervention. Front Endocrinol. (2021) 12:12. doi: 10.3389/fendo.2021.710240, PMID: 34489866 PMC8417234

[8] Zhao Q Cheng Y-J Xu Y-K Zhao Z-W Liu C Sun T-N. Comparison of various insulin resistance surrogates on prognostic prediction and stratification following percutaneous coronary intervention in patients with and without type 2 diabetes mellitus. Cardiovasc Diabetol. (2021) 20:190. doi: 10.1186/s12933-021-01383-7, PMID: 34537077 PMC8449896

[9] Chen C-l Liu L Lo K Huang J-y Yu Y-l Huang Y-q. Association between triglyceride glucose index and risk of new-onset diabetes among Chinese adults: findings from the China health and retirement longitudinal study. Front Cardiovasc Med. (2020) 7:7. doi: 10.3389/fcvm.2020.610322, PMID: 33330672 PMC7728664

[10] Shi W Qin M Wu S Xu K Zheng Q Liu X. Usefulness of triglyceride-glucose index for detecting prevalent atrial fibrillation in a type 2 diabetic population. Postgrad Med. (2022) 134:820–8. doi: 10.1080/00325481.2022.2124088, PMID: 36093727

[11] Kosmas CE Bousvarou MD Kostara CE Papakonstantinou EJ Salamou E Guzman E. Insulin resistance and cardiovascular disease. J Int Med Res. (2023) 51:3000605231164548. doi: 10.1177/03000605231164548, PMID: 36994866 PMC10069006

[12] Deng X-L Liu Z Wang C Li Y Cai Z. Insulin resistance in ischemic stroke. Metab Brain Dis. (2017) 32:1323–34. doi: 10.1007/s11011-017-0050-0 28634787

[13] Jing J Pan Y Zhao X Zheng H Jia Q Mi D. Insulin resistance and prognosis of nondiabetic patients with ischemic stroke. Stroke. (2017) 48:887–93. doi: 10.1161/strokeaha.116.015613, PMID: 28235959

[14] Chang Y Kim CK Kim M-K Seo WK Oh K. Insulin resistance is associated with poor functional outcome after acute ischemic stroke in non-diabetic patients. Sci Rep. (2021) 11:1229. doi: 10.1038/s41598-020-80315-z, PMID: 33441784 PMC7806587

[15] Ago T Matsuo R Hata J Wakisaka Y Kuroda J Kitazono T. Insulin resistance and clinical outcomes after acute ischemic stroke. Neurology. (2018) 90:e1470–7. doi: 10.1212/wnl.0000000000005358 29602916

[16] Strisciuglio T Izzo R Barbato E Di Gioia G Colaiori I Fiordelisi A. Insulin resistance predicts severity of coronary atherosclerotic disease in non-diabetic Patients. J Clin Med. (2020) 9:2144. doi: 10.3390/jcm9072144, PMID: 32646007 PMC7408744

[17] Vasques ACJ Novaes FS de Oliveira MS Matos Souza JR Yamanaka A Pareja JC. TyG index performs better than HOMA in a Brazilian population: a hyperglycemic clamp validated study. Diabetes Res Clin Pract. (2011) 93:e98–e100. doi: 10.1016/j.diabres.2011.05.030, PMID: 21665314

[18] Pan Y Jing J Chen W Zheng H Jia Q Mi D. Post-glucose load measures of insulin resistance and prognosis of nondiabetic patients with ischemic stroke. J Am Heart Assoc. (2017) 6:e004990. doi: 10.1161/jaha.116.004990, PMID: 28108466 PMC5523645

[19] Wang S Shi J Peng Y Fang Q Mu Q Gu W. Stronger association of triglyceride glucose index than the HOMA-IR with arterial stiffness in patients with type 2 diabetes: a real-world single-Centre study. Cardiovasc Diabetol. (2021) 20:82. doi: 10.1186/s12933-021-01274-x, PMID: 33888131 PMC8063289

[20] Hribal ML Er L-K Wu S Chou H-H Hsu L-A Teng M-S. Triglyceride glucose-body mass index is a simple and clinically useful surrogate marker for insulin resistance in nondiabetic individuals. PLoS One. (2016) 11:e0149731. doi: 10.1371/journal.pone.0149731, PMID: 26930652 PMC4773118

[21] Kahn BB Flier JS. Obesity and insulin resistance. J Clin Invest. (2000) 106:473–81. doi: 10.1172/jci10842, PMID: 10953022 PMC380258

[22] Wang X Liu J Cheng Z Zhong Y Chen X Song W. Triglyceride glucose-body mass index and the risk of diabetes: a general population-based cohort study. Lipids Health Dis. (2021) 20:99. doi: 10.1186/s12944-021-01532-7, PMID: 34488806 PMC8420033

[23] Li Y Gui J Liu H Guo LL Li J Lei Y. Predicting metabolic syndrome by obesity-and lipid-related indices in mid-aged and elderly Chinese: a population-based cross-sectional study. Front Endocrinol. (2023) 14:1201132. doi: 10.3389/fendo.2023.1201132, PMID: 37576971 PMC10419183

[24] Raimi TH Dele-Ojo BF Dada SA Fadare JO Ajayi DD Ajayi EA. Triglyceride-glucose index and related parameters predicted metabolic syndrome in Nigerians. Metab Syndr Relat Disord. (2021) 19:76–82. doi: 10.1089/met.2020.0092, PMID: 33170086 PMC7929914

[25] Zhou M Wang H Zeng X Yin P Zhu J Chen W. Mortality, morbidity, and risk factors in China and its provinces, 1990–2017: a systematic analysis for the global burden of disease study 2017. Lancet. (2019) 394:1145–58. doi: 10.1016/s0140-6736(19)30427-1, PMID: 31248666 PMC6891889

[26] Virani SS Alonso A Benjamin EJ Bittencourt MS Callaway CW Carson AP. Heart disease and stroke statistics—2020 update: a report from the American Heart Association. Circulation. (2020) 141:e139–596. doi: 10.1161/cir.0000000000000757, PMID: 31992061

[27] Kim JS Caplan LR. Clinical stroke syndromes. Intracranial atherosclerosis: pathophysiology, diagnosis and treatment. Front Neurol Neurosci. (2016) 40:72–92. doi: 10.1159/000448303 27960164

[28] Wang W Jiang B Sun H Ru X Sun D Wang L. Prevalence, incidence, and mortality of stroke in China. Circulation. (2017) 135:759–71. doi: 10.1161/circulationaha.116.025250, PMID: 28052979

[29] Lu J Lu Y Wang X Li X Linderman GC Wu C. Prevalence, awareness, treatment, and control of hypertension in China: data from 1·7 million adults in a population-based screening study (China PEACE million persons project). Lancet. (2017) 390:2549–58. doi: 10.1016/s0140-6736(17)32478-9 29102084

[30] Liu T Xuan H Yin J Wang L Wang C Xu X. Triglyceride glucose index increases significantly risk of hypertension development in Chinese individuals aged ≥45 years old: analysis from the China health and retirement longitudinal study. J Multidiscip Healthc. (2023) 16:63–73. doi: 10.2147/jmdh.S391905, PMID: 36660037 PMC9842521

[31] Zhao Y Hu Y Smith JP Strauss J Yang G. Cohort profile: the China health and retirement longitudinal study (CHARLS). Int J Epidemiol. (2014) 43:61–8. doi: 10.1093/ije/dys203, PMID: 23243115 PMC3937970

[32] Unger T Borghi C Charchar F Khan NA Poulter NR Prabhakaran D. International Society of Hypertension global hypertension practice guidelines. J Hypertens. (2020, 2020) 38:982–1004. doi: 10.1097/hjh.0000000000002453 32371787

[33] Joint Committee for Guideline Revision. 2018 Chinese guidelines for prevention and treatment of hypertension-a report of the revision Committee of Chinese Guidelines for prevention and treatment of hypertension. J Geriatr Cardiol. (2019) 16:182–241. doi: 10.11909/j.issn.1671-5411.2019.03.014, PMID: 31080465 PMC6500570

[34] Huo R-R Liao Q Zhai L You X-M Zuo Y-L. Interacting and joint effects of triglyceride-glucose index (TyG) and body mass index on stroke risk and the mediating role of TyG in middle-aged and older Chinese adults: a nationwide prospective cohort study. Cardiovasc Diabetol. (2024) 23:30. doi: 10.1186/s12933-024-02122-4, PMID: 38218819 PMC10790273

[35] Li H Zheng D Li Z Wu Z Feng W Cao X. Association of Depressive Symptoms with Incident Cardiovascular Diseases in middle-aged and older Chinese adults. JAMA Netw Open. (2019) 2:e1916591. doi: 10.1001/jamanetworkopen.2019.16591, PMID: 31800066 PMC6902756

[36] Xie W Liang L Zhao L Shi P Yang Y Xie G. Combination of carotid intima-media thickness and plaque for better predicting risk of ischaemic cardiovascular events. Heart. (2011) 97:1326–31. doi: 10.1136/hrt.2011.223032, PMID: 21653216

[37] Qiao Q Liang K Wang C Wang L Yan F Chen L. J-shaped association of the triglyceride glucose-body mass index with new-onset diabetes. Sci Rep. (2024) 14:13882. doi: 10.1038/s41598-024-64784-0, PMID: 38880800 PMC11180648

[38] Shao Y Hu H Li Q Cao C Liu D Han Y. Link between triglyceride-glucose-body mass index and future stroke risk in middle-aged and elderly Chinese: a nationwide prospective cohort study. Cardiovasc Diabetol. (2024) 23:81. doi: 10.1186/s12933-024-02165-7, PMID: 38402161 PMC10893757

[39] Zhu Y Li Y Li X Huang S Li Y. Association between triglyceride glucose-body mass index and all-cause mortality in critically ill patients with acute pancreatitis. Sci Rep. (2024) 14:21605. doi: 10.1038/s41598-024-72969-w, PMID: 39285256 PMC11405403

[40] Chen T Wan H Luo Y Chen L. Association of triglyceride-glucose-body mass index with all-cause and cardiovascular mortality among individuals with chronic kidney disease. Sci Rep. (2024) 14:20593. doi: 10.1038/s41598-024-71579-w, PMID: 39232126 PMC11375041

[41] Zeng ZY Liu SX Xu H Xu X Liu XZ Zhao XX. Association of triglyceride glucose index and its combination of obesity indices with prehypertension in lean individuals: a cross-sectional study of Chinese adults. J Clin Hypertens. (2020) 22:1025–32. doi: 10.1111/jch.13878, PMID: 32442359 PMC8029919

[42] Wang R Dai L Zhong Y Xie G. Usefulness of the triglyceride glucose-body mass index in evaluating nonalcoholic fatty liver disease: insights from a general population. Lipids Health Dis. (2021) 20:77. doi: 10.1186/s12944-021-01506-9, PMID: 34321005 PMC8317400

[43] Forouzanfar MH Afshin A Alexander LT Anderson HR Bhutta ZA Biryukov S. Global, regional, and national comparative risk assessment of 79 behavioural, environmental and occupational, and metabolic risks or clusters of risks, 1990–2015: a systematic analysis for the global burden of disease study 2015. Lancet. (2016) 388:1659–724. doi: 10.1016/s0140-6736(16)31679-8 27733284 PMC5388856

[44] Oparil S Acelajado MC Bakris GL Berlowitz DR Cífková R Dominiczak AF. Hypertension. Nat Rev Dis Primers. (2018) 4:1. doi: 10.1038/nrdp.2018.14, PMID: 29565029 PMC6477925

[45] Nikbakht HR Najafi F Shakiba E Darbandi M Navabi J Pasdar Y. Triglyceride glucose-body mass index and hypertension risk in iranian adults: a population-based study. BMC Endocr Disord. (2023) 23:156. doi: 10.1186/s12902-023-01411-5, PMID: 37479987 PMC10360216

[46] Deng D Chen C Wang J Luo S Feng Y. Association between triglyceride glucose-body mass index and hypertension in Chinese adults: a cross-sectional study. J Clin Hypertens. (2023) 25:370–9. doi: 10.1111/jch.14652, PMID: 36929716 PMC10085812

[47] Dimitriadis G Mitrou P Lambadiari V Maratou E Raptis SA. Insulin effects in muscle and adipose tissue. Diabetes Res Clin Pract. (2011) 93:S52–9. doi: 10.1016/s0168-8227(11)70014-6 21864752

[48] Ormazabal V Nair S Elfeky O Aguayo C Salomon C Zuñiga FA. Association between insulin resistance and the development of cardiovascular disease. Cardiovasc Diabetol. (2018) 17:122. doi: 10.1186/s12933-018-0762-4, PMID: 30170598 PMC6119242

[49] Reaven GM. Pathophysiology of insulin resistance in human disease. Physiol Rev. (1995) 75:473–86. doi: 10.1152/physrev.1995.75.3.473 7624391

[50] Wilcox G. Insulin and insulin resistance. Clin Biochem Rev. (2005) 26:19–39. PMID: 16278749 PMC1204764

[51] Xie Y Guo R Li Z Guo X Sun G Sun Z. Temporal relationship between body mass index and triglyceride-glucose index and its impact on the incident of hypertension. Nutr Metab Cardiovasc Dis. (2019) 29:1220–9. doi: 10.1016/j.numecd.2019.07.003, PMID: 31383505

[52] Salvetti A Brogi G Di Legge V Bernini GP. The inter-relationship between insulin resistance and hypertension. Drugs. (1993) 46:149–59. doi: 10.2165/00003495-199300462-00024, PMID: 7512468

[53] Tsao CW Aday AW Almarzooq ZI Alonso A Beaton AZ Bittencourt MS. Heart disease and stroke statistics—2022 update: a report from the American Heart Association. Circulation. (2022) 145:e153–639. doi: 10.1161/cir.0000000000001052, PMID: 35078371

[54] Choe S Lee J Lee J Kang D Lee J-K Shin A. Validity of self-reported stroke and myocardial infarction in Korea: the health examinees (HEXA) study. J Prev Med Public Health. (2019) 52:377–83. doi: 10.3961/jpmph.19.089, PMID: 31795614 PMC6893227

[55] Yuan X Liu T Wu L Zou ZY Li C. Validity of self-reported diabetes among middle-aged and older Chinese adults: the China health and retirement longitudinal study. BMJ Open. (2015) 5:e006633. doi: 10.1136/bmjopen-2014-006633, PMID: 25872937 PMC4401856

