# Supplementary material for: Combined effect of triglyceride glucose-body mass index and hypertension on new-onset stroke: evidence from the China health and retirement longitudinal study
Source: Front Public Health. 2024 Oct 25;12:1432742. doi: 10.3389/fpubh.2024.1432742 (PMC11543489; doi:10.3389/fpubh.2024.1432742)
Supplement: Supplementary file 1 [file Data_Sheet_1.docx]

Supplementary Material

**Combined effect of triglyceride glucose****-body mass index and hypertension on new-onset stroke: evidence from the China Health and Retirement Longitudinal Study (CHARLS)**

**Fucun Ma^1^, Jiaying Hu^1^, Zheng Gao^1^, Xuekai Liu^1^, Mingjian Bai^1*^, Guowei Liang^1*^**

^1^Department of Clinical Laboratory, Aerospace Center Hospital, Beijing 100049, China

(Guowei Liang and Mingjian Bai contributed to this work equally and should be considered as co-corresponding authors)

*** Correspondence:**

Corresponding Author: Guowei Liang

Email: [LGW721@163.com](mailto:LGW721@163.com)

# Supplementary Tables

**Table S1.** Association of TyG-BMI levels and new-onset stroke in CAHRLS (2011-2018)

| New-onset stroke | Group 1 | Group 2 |
| --- | --- | --- |
| Cases, n (%) | 155/3,461(4.48) | 246/3,461(7.11) |
| Model 1^a^ | 1.00(ref.) | 1.74(1.41-2.14) |
| Model 2^b^ | 1.00(ref.) | 1.43(1.10-1.87) |
| Model 3^c^ | 1.00(ref.) | 1.37(1.05-1.80) |

Group 1 is TyG-BMI <199.74; Group 2 is TyG-BMI ≥199.74

^a^Model 1 adjusted for age, sex;

^b^Model 2 further adjusted for further adjusting for education level, marital status, household status, drinking status, smoking status, FBG, waist measurement, TG, CRP and BMI based on model 1.

^c^Model 3 further adjusted for diabetes mellitus, cancer, arthritis, dyslipidemia, digestive disease, lung disease, psych problem, liver disease, kidney disease and memory problem based on model 2.

**Table S2.** Association of hypertension levels and new-onset stroke in CAHRLS (2011-2018)

| New-onset stroke | Group 1 | Group 2 |
| --- | --- | --- |
| Cases, n (%) | 143/4,177(3.42) | 258/2,745(9.40) |
| Model 1^a^ | 1.00(ref.) | 2.58(2.09-3.18) |
| Model 2^b^ | 1.00(ref.) | 2.33(1.87-2.91) |
| Model 3^c^ | 1.00(ref.) | 2.22(1.77-2.78) |

Group 1 is non-hypertension; Group 2 is hypertension

^a^Model 1 adjusted for age, sex;

^b^Model 2 further adjusted for further adjusting for education level, marital status, household status, drinking status, smoking status, FBG, waist measurement, TG, CRP and BMI based on model 1.

^c^Model 3 further adjusted for diabetes mellitus, cancer, arthritis, dyslipidemia, digestive disease, lung disease, psych problem, liver disease, kidney disease and memory problem based on model 2.

**Table S3.** TyG-BMI Index Association with new-onset cardiovascular diseases in CHARLS (2011-2018)

| New-onset CVD | Group1 | Group2 | Group3 | Group4 | *P* trend |
| --- | --- | --- | --- | --- | --- |
| Case, n (%) | 154(10.05) | 215(14.03) | 232(15.15) | 303(19.77) |  |
| Model 1^a^ | 1.00(ref.) | 1.45(1.18-1.79) | 1.59(1.29-1.95) | 2.16(1.77-2.64) | <0.001 |
| Model 2^b^ | 1.00(ref.) | 1.35(1.09-1.67) | 1.37(1.09-1.71) | 1.68(1.31-2.15) | <0.001 |
| Model 3^c^ | 1.00(ref.) | 1.41(1.13-1.75) | 1.43(1.13-1.79) | 1.68(1.30-2.16) | <0.001 |

^a^Model 1 adjusted for age, sex;

^b^Model 2 further adjusted for further adjusting for education level, marital status, household status, drinking status, smoking status, waist measurement, CRP and hypertension based on model 1.

^c^Model 3 further adjusted for diabetes mellitus, cancer, arthritis, dyslipidemia, digestive disease, lung disease, psych problem, liver disease, kidney disease and memory problem based on model 2.

**Table S4.** TyG-BMI Index Association with new-onset diabetes in CHARLS (2011-2018)

| New-onset diabetes | Group1 | Group2 | Group3 | Group4 | *P* trend |
| --- | --- | --- | --- | --- | --- |
| Case, n (%) | 74(3.96) | 118(6.31) | 182(9.74) | 375(20.05) |  |
| Model 1^a^ | 1.00(ref.) | 1.68(1.23-2.29) | 2.54(1.90-3.41) | 5.72(4.37-7.50) | <0.001 |
| Model 2^b^ | 1.00(ref.) | 1.61(1.17-2.20) | 2.29(1.69-3.12) | 4.80(3.49-6.59) | <0.001 |
| Model 3^c^ | 1.00(ref.) | 1.64(1.19-2.27) | 2.39(1.74-3.27) | 4.91(3.54-6.81) | <0.001 |

^a^Model 1 adjusted for age, sex;

^b^Model 2 further adjusted for further adjusting for education level, marital status, household status, drinking status, smoking status, waist measurement, CRP and hypertension based on model 1.

^c^Model 3 further adjusted for diabetes mellitus, cancer, arthritis, dyslipidemia, digestive disease, lung disease, psych problem, liver disease, kidney disease and memory problem based on model 2.

**Table S5.** Sensitivity analysis considering different definition of hypertension for association of TyG-BMI index and hypertension^*^ with new-onset stroke in CAHRLS (2011-2018)

| New-onset stroke | Group 1 | Group 2 | Group 3 | Group 4 |
| --- | --- | --- | --- | --- |
| Cases, n (%) | 70/2,450(2.86) | 73/1,727(4.23) | 85/1,011(8.41) | 173/1,734(9.98) |
| Model 1^a^ | 1.00(ref.) | 1.62(1.16-2.26) | 2.67(1.93-3.69) | 3.49(2.63-4.63) |
| Model 2^b^ | 1.00(ref.) | 1.49(1.04-2.15) | 2.63(1.90-3.65) | 3.05(2.17-4.28) |
| Model 3^c^ | 1.00(ref.) | 1.49(1.03-2.16) | 2.60(1.87-3.61) | 2.80(1.99-3.96) |

^*^Hypertension was defined as SBP at least 130 mmHg, DBP at least 80 mmHg, a self-reported history of hypertension, with antihypertensive drug, Group 1 is TyG-BMI <199.74 and non-hypertension; Group 2 is TyG-BMI ≥199.74 and non-hypertension; Group 3 is TyG-BMI <199.74 and hypertension; Group 4 is TyG-BMI ≥199.74 and hypertension.

^a^Model 1 adjusted for age, sex;

^b^Model 2 further adjusted for further adjusting for education level, marital status, household status, drinking status, smoking status, FBG, waist measurement, TG, CRP and BMI based on model 1.

^c^Model 3 further adjusted for diabetes mellitus, cancer, arthritis, dyslipidemia, digestive disease, lung disease, psych problem, liver disease, kidney disease and memory problem based on model 2.

**Table S6.** Sensitivity analysis considering medications history for association of TyG-BMI index and hypertension with new-onset stroke in CHARLS (2011-2018)

| New-onset stroke | Group1 | Group2 |  | Group3 | Group4 |
| --- | --- | --- | --- | --- | --- |
| Case, n (%) | 70(2.86) | 73(4.23) |  | 85(8.41) | 173(9.98) |
| Model 1^a^ | 1.00(ref.) | 1.62(1.16-2.26) |  | 2.67(1.93-3.69) | 3.49(2.63-4.63) |
| Model 2^b^ | 1.00(ref.) | 1.49(1.04-2.15) |  | 2.63(1.90-3.65) | 3.05(2.17-4.28) |
| Model 3^c^ | 1.00(ref.) | 1.49(1.03-2.16) |  | 2.60(1.87-3.61) | 2.81(1.99-3.96) |
| Model 4^d^ | 1.00(ref.) | 1.50(1.04-2.17) |  | 2.31(1.63-3.26) | 2.38(1.64-3.46) |

Group 1 is TyG-BMI <199.74 and non-hypertension; Group 2 is TyG-BMI ≥199.74 and non-hypertension; Group 3 is TyG-BMI <199.74 and hypertension; Group 4 is TyG-BMI ≥199.74 and hypertension.

^a^Model 1 adjusted for age, sex;

^b^Model 2 further adjusted for further adjusting for education level, marital status, household status, drinking status, smoking status, FBG, waist measurement, TG, CRP and BMI based on model 1.

^c^Model 3 further adjusted for diabetes mellitus, cancer, arthritis, dyslipidemia, digestive disease, lung disease, psych problem, liver disease, kidney disease and memory problem based on model 2.

^d^Model 4 further adjusted for taking medications for hypertension, diabetes and dyslipidemia based on model 3.

**Table S7.** Sensitivity analysis excluded treated hypertension patients^*^ (N=1,223) for association of TyG-BMI index and hypertension with new-onset stroke in CAHRLS (2011-2018)

| New-onset stroke | Group 1 | Group 2 | Group 3 | Group 4 |
| --- | --- | --- | --- | --- |
| Cases, n (%) | 70/2,450(2.86) | 73/1,727(4.23) | 49/682(7.18) | 69/840(8.21) |
| Model 1^a^ | 1.00(ref.) | 1.63(1.17-2.28) | 2.17(1.49-3.16) | 2.91(2.08-4.09) |
| Model 2^b^ | 1.00(ref.) | 1.48(0.99-2.22) | 2.14(1.47-3.33) | 2.47(1.61-3.79) |
| Model 3^c^ | 1.00(ref.) | 1.46(0.97-2.19) | 2.15(1.47-3.16) | 2.35(1.53-3.61) |

^*^is sensitivity analysis with excluded treated hypertension patients (N=1,223)

Group 1 is TyG-BMI <199.74 and non-hypertension; Group 2 is TyG-BMI ≥199.74 and non-hypertension; Group 3 is TyG-BMI <199.74 and hypertension; Group 4 is TyG-BMI ≥199.74 and hypertension.

^a^Model 1 adjusted for age, sex;

^b^Model 2 further adjusted for further adjusting for education level, marital status, household status, drinking status, smoking status, FBG, waist measurement, TG, CRP and BMI based on model 1.

^c^Model 3 further adjusted for diabetes mellitus, cancer, arthritis, dyslipidemia, digestive disease, lung disease, psych problem, liver disease, kidney disease and memory problem based on model 2.

## Supplementary Figures


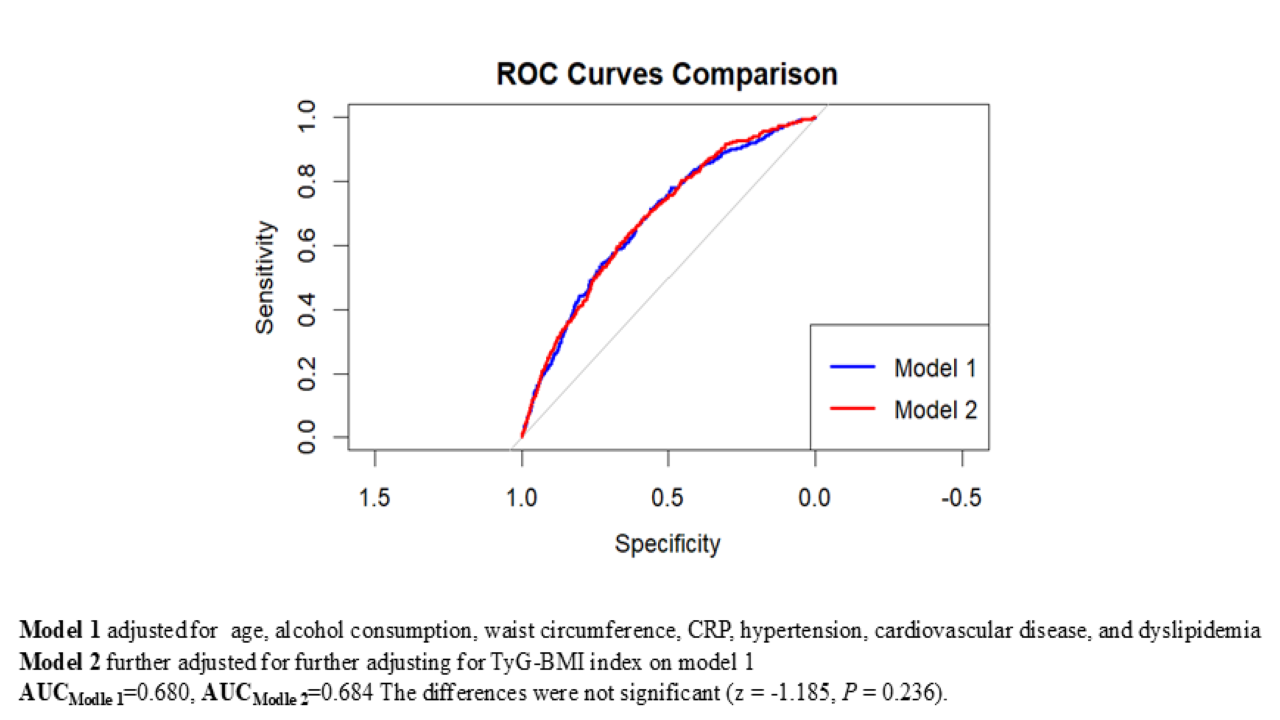


**Supplementary Figure S1.** ROC curve analysis comparing the discriminatory ability of models with and without TyG-BMI for predicting stroke events


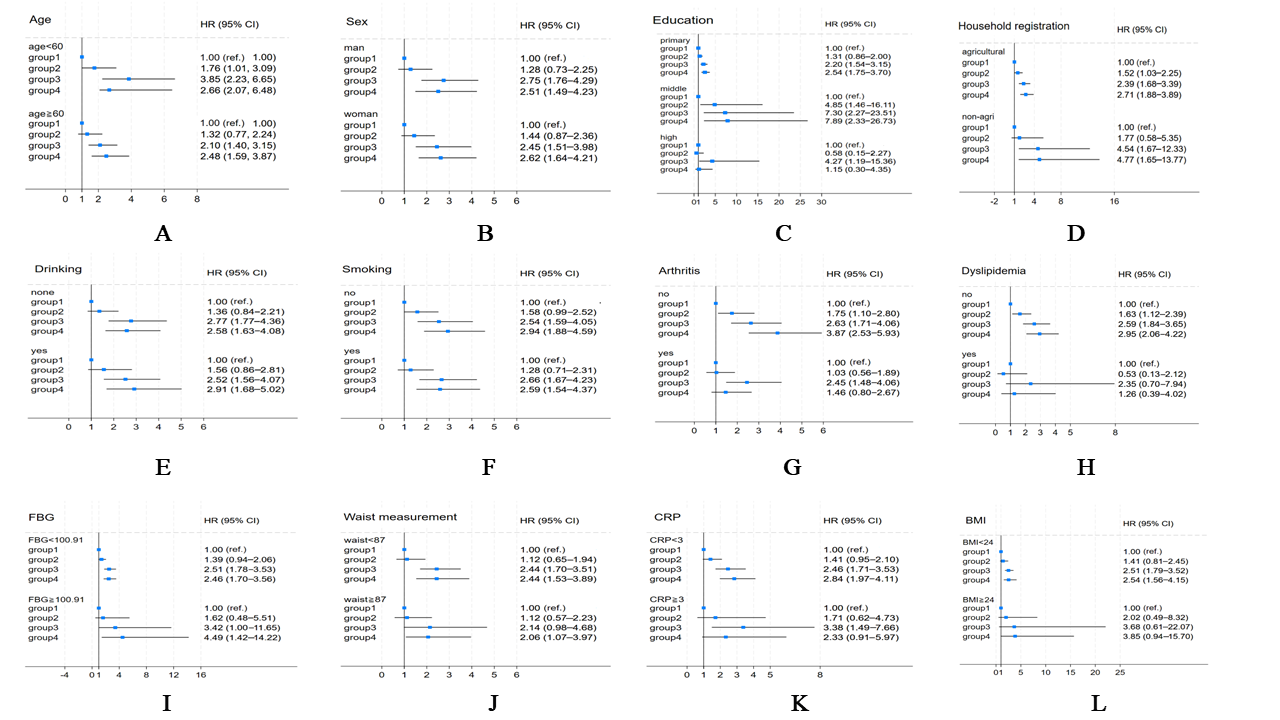


**Supplementary Figure** **S2**. Subgroup analyses of the association of elevated TyG-BMI levels and hypertension with new-onset stroke in CHARLS.
